# TRPM2 in ischemic stroke: Structure, molecular mechanisms, and drug intervention

**DOI:** 10.1080/19336950.2020.1870088

**Published:** 2021-01-18

**Authors:** Qing Wang, Ning Liu, Yuan-Shu Ni, Jia-Mei Yang, Lin Ma, Xiao-Bing Lan, Jing Wu, Jian-Guo Niu, Jian-Qiang Yu

**Affiliations:** a Department of Pharmacology, Ningxia Medical University, Yinchuan, China; b Ningxia Key Laboratory of Craniocerebral Diseases of Ningxia Hui Autonomous Region, Ningxia Medical University, Yinchuan, China; c Laboratory Animal Center, Ningxia Medical University, Yinchuan, China; d Ningxia Collaborative Innovation Center of Regional Characteristic Traditional Chinese Medicine, Ningxia Medical University, Yinchuan, Ningxia, China

**Keywords:** TRPM2 channel, ischemic stroke, pathogenesis, TRPM2 blockers, virtual screening

## Abstract

Ischemic stroke has a high lethality rate worldwide, and novel treatments are limited. Calcium overload is considered to be one of the mechanisms of cerebral ischemia. Transient receptor potential melastatin 2 (TRPM2) is a reactive oxygen species (ROS)-sensitive calcium channel. Cerebral ischemia-induced TRPM2 activation triggers abnormal intracellular Ca^2+^ accumulation and cell death, which in turn causes irreversible brain damage. Thus, TRPM2 has emerged as a new therapeutic target for ischemic stroke. This review provides data on the expression, structure, and function of TRPM2 and illustrates its cellular and molecular mechanisms in ischemic stroke. Natural and synthetic TRPM2 inhibitors (both specific and nonspecific) are also summarized. The three-dimensional protein structure of TRPM2 has been identified, and we speculate that molecular simulation techniques will be essential for developing new drugs that block TRPM2 channels. These insights about TRPM2 may be the key to find potent therapeutic approaches for the treatment of ischemic stroke.

## Introduction

Stroke is a global neurological condition with both high morbidity and mortality and is the leading cause of long-term disability worldwide. Ischemic stroke accounts for approximately 85% of all stroke cases [1,2]. Long-term ischemia can lead to fatal brain damage. At present, available emergency procedures aim to restore blood circulation as soon as possible following ischemia. However, reestablishing perfusion in the infarcted area results in the more severe damage and deterioration of ischemic brain tissue. Currently, emerging therapies for stroke based on intravenous and intra-arterial therapies are confined with their efficacy, and only a small proportion of patients are eligible to receive these therapies due to the narrow therapeutic time window [3]. Therefore, a novel, safe, and efficient treatment option is still necessary for ischemic stroke.

The energy requirements of the brain are quite high relative to other organs. Ischemic brain injury initially leads to the excessive consumption of high-energy phosphates, particularly adenosine triphosphate (ATP) and phosphocreatine [4]. Neurons qare unable to sustain their normal transmembrane ionic gradient and homeostasis [5]. The ATP-dependent Ca^2+^ pumps lead to a dramatic rise in Ca^2+^ during ischemia. Calcium is known to be an essential second messenger involved in related physiological functions. Excessive increases of intracellular calcium ions cause glutamate release, mitochondrial dysfunction, oxidative stress, inflammation, and other detrimental cascades [6,7]. These pathophysiological processes are seriously injurious to neurons, glia, and endothelial cells [8–11], which forms a positive feedback loop and causes brain destruction [12]. Taken together, ischemia leads to calcium overload and cell death.

Recently, emerging evidence has revealed that transient receptor potential melastatin 2 (TRPM2), also called LTRPC-2 [13] or TRPC7 [14], the most abundant transient receptor potential (TRP) channel, is a Ca^2+^-permeable, nonselective cation channel [14,15]. TRPM2 is highly distributed in the central nervous system and is activated by hydrogen peroxide (H_2_O_2_) and agents that produce reactive oxygen/nitrogen species (ROS/RNS), increasing the Ca^2+^ concentration [16,17]. A growing number of studies have consistently revealed that TRPM2 is detrimental in brain ischemia [18–20]. Meanwhile, inhibiting endogenous TRPM2 suppressed Ca^2+^ influx and cell death induced by ischemic brain injury in vivo and in vitro [21–23]. Therefore, modulation of TRPM2 can be a potential therapeutic strategy to prevent ischemia-induced neuronal death. Here, we will discuss the distribution, expression, structure, and activation of TRPM2 with a focus on the underlying molecular mechanisms in ischemic stroke pathogenesis. We also present recent progress and challenges in potential treatments targeting TRPM2 in ischemic stroke.

## Distribution and expression of TRPM2 in the brain

Previous studies demonstrated that TRPM2 expression was abundant in the central nervous system (CNS) and was associated with neurodegenerative disease including ischemic stroke. Indeed, TRPM2 is expressed in neurons in the hippocampus [24], cortex [25], striatum [26], and substantia nigra [27]. Olah et al. observed that TRPM2 was highly expressed in the hippocampus’ pyramidal neurons, including the CA1 region, by using biochemical and molecular approaches [24]. The majority of glial cells in the CNS are mainly microglia, astrocytes, and oligodendrocytes. As the CNS resident macrophages, microglia maintain cellular homeostasis via the clearance of old synapses or other debris. A profile of TRPM2 expression in a panel of human cell-lines and purified cells revealed substantial expression in the C13 microglial line [28]. Meanwhile, the study has revealed that the expression of TRPM2 was in stellate neurons of the mouse ventral cochlear nucleus (VCN) [29] and highly enriched in astrocytes [30]. However, relying on cell lines only is likely to be unreliable and this needs confirmation in intact human tissue. The blood-brain barrier (BBB) dysfunction-induced inflammatory signaling is a crucial pathophysiologic factor in stroke development. The BBB is primarily composed of endothelial cells, astrocytes, and pericytes [31]. Recently, TRPM2 has been found to be expressed in neurovascular endothelial cells [32] and pericytes [33]. To summarize, TRPM2 is widely distributed in the CNS.

## TRPM2 channel structure and activation

**Figure 1.**
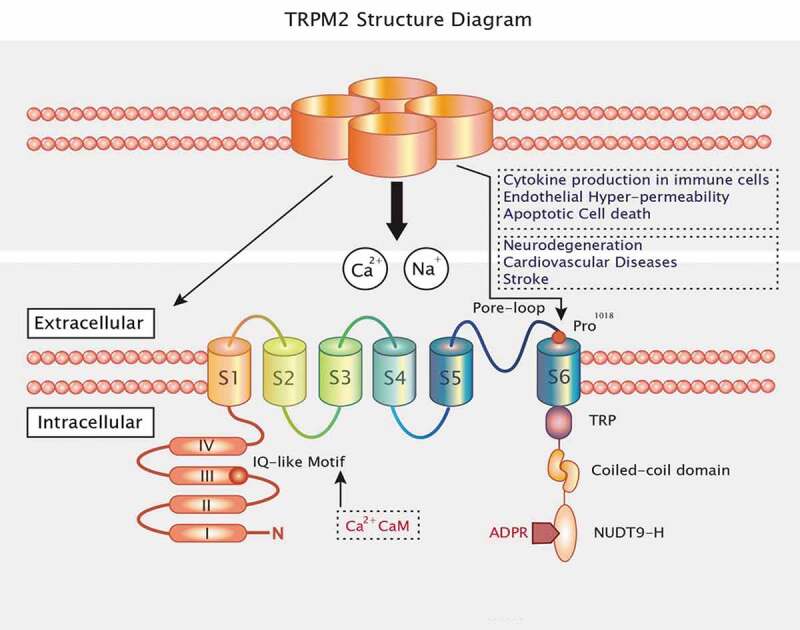
TRPM2 consists of four subunits and has six transmembrane domains with a reentry loop between the fifth and sixth helices. The N termini and C termini are located in the intracellular loops. The intracellular N-terminus includes four highly conserved common regions and an IQ motif that binds CaM and Ca^2+^. The intracellular C-terminus contains a TRP box (TRP), a coiled-coil domain (CCD), and the nucleoside diphosphate-linked moiety X-type homology motif (NUDT9-H)

The human TRPM2 gene is generally located on chromosome 21q22.3 and encodes 1503 amino acid residues [34]. Molecularly, TRPM2 includes four identical subunits and six transmembrane domains (S1–S6). It has a pore-forming reentry loop region between domains 5 and 6. TRPM2 proteins are composed of cytoplasmic N- and C-terminal regulatory domains [13,35]. On the one hand, the N-terminus comprises four TRPM channel family homologous members and a calmodulin (CaM)-binding IQ motif located at residues 404–416, which contributes to TRPM2 activation based on intracellular calcium [36]. On the other hand, TRPM2 has a TRP segment at the C-terminal transmembrane domain, divided into two regions: a second variable region and a coiled-coil domain. In TRPM2 C-terminus, the coiled-coiled motif interacts with specific subunits and assembles TRPM2 into its functional tetrameric form [37]. Moreover, the C-terminus of TRPM2 also contains a nucleoside diphosphate-linked X-type homology motif (NUDT9-H) that includes an 11-residue ADP-ribose (ADPR) binding pocket [13,38]. ADPR has been thought of as a novel second messenger regulating Ca^2+^ influx, and to gait TRPM2 opening directly [39] (Figure 1).

**Figure 2.**
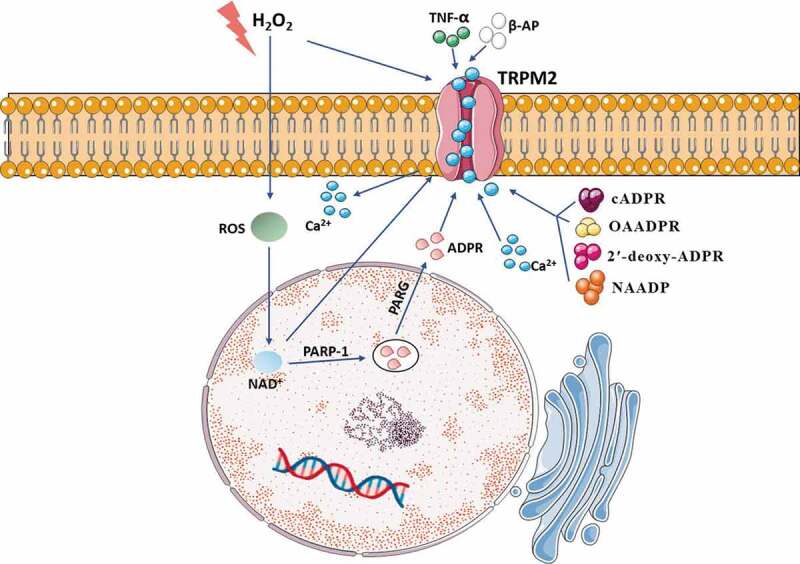
Various stimuli can lead to TRPM2 activation. The elevation of ROS and H_2_O_2_ activate TRPM2 through production of intracellular ADPR. Extracellular factors including TNF-α and β-AP contribute to activation of TRPM2. Structural analogs of ADPR including NAADP, cADPR, OAADPR, and 2′-deoxy-ADPR activate TRPM2. Elevated Ca^2+^ and NAD^+^ can also participate in activation of TRPM2

The study has proposed that TRPM2 sensitizes HEK 293 cells to H_2_O_2_-induced cell death, which strongly supports that TRPM2 mediates cell death as an endogenous calcium-permeable channel following severe oxidative stress [17]. There are several pathways that can activate TRPM2: (1) The elevation of ROS results in activation of the poly-ADPR polymerase (PARP) and poly-ADPR glycohydrolase (PARG) and Deoxyribose Nucleic Acid (DNA) damage. Then, PARP and PARG turn nicotinamide adenine dinucleotide (NAD^+^) into ADPR monomers to activate TRPM2 [40,41]. (2) Besides, some of these extracellular factors, including H_2_O_2_ [16], tumor necrosis factor-α (TNF-α) [42], β-amyloid peptide (β-AP) [43] activate TRPM2 through the generation of intracellular ADPR. (3) H_2_O_2_ and NAD^+^ can activate TRPM2 directly [17,39,44]. (4) TRPM2 has also been reported to be activated by structural analogs of ADPR, including nicotinic acid adenine dinucleotide phosphate (NAADP), cyclic ADPR (cADPR), 2′-O-acetyl-ADPR (OAADPR), and 2′-deoxy-ADPR [45,46]. (5) Cellular Ca^2+^ is involved in multiple functions. Ca^2+^ interacts with TRPM2 at the IQ-like motif in the N terminus, contributing to ADPR-mediated TRPM2 activation [36,47]. Moreover, the level of Ca^2+^ can significantly decrease the ADPR concentration required for TRPM2 activation [13]. (6) TRPM2-S inhibits intracellular calcium influx, and TRPM2-L exposed to H_2_O_2_ reduces cell viability. Therefore, TRPM2 splicing regulation is essential to cell apoptosis [48]. As is introduced above, TRPM2 is activated by oxidative stress and other intracellular pathways (Figure 2).

## Effects of TRPM2 on the pathogenesis of cerebral ischemia

In recent years, growing evidence has provided insight that TRPM2 played prominent roles in cellular damage and mediated brain injury in ischemic stroke due to their sensitivity to oxidative stress. In the disease model of ischemic stroke, TRPM2 mRNA levels were significantly elevated [28]. Also, TRPM2 leads to oxidative stress-induced cell damage [49]. Following this logic, we propose that TRPM2 can be a potential target for preventing ischemic stroke. Therefore, in the following sections, we summarize recent research progress toward understanding the causes and consequences of pathological TRPM2 activation associated with ischemic stroke.

### Mechanisms of neuronal TRPM2 in delayed cell death

Based on in vitro and in vivo models of ischemic stroke, different neuronal molecular mechanisms might be responsible for the allosteric regulation of TRPM2. During a period of acute ischemia in vivo or oxygen-glucose deprivation (OGD) in vitro, TRPM2 is activated by cellular stress and contributes to ischemia-induced membrane depolarization, intracellular calcium accumulation, and cell swelling [50]. Interestingly, Verma et al. observed a sex-specific protective role for TRPM2 in cortical and hippocampal neuronal response in vitro OGD using TRPM2 inhibitors or small hairpin RNA (shRNA)-mediated knockdown of TRPM2 [19]. Similarly, Jia et al. observed that TRPM2 inhibition with Clotrimazole and shRNA virus decreased infarct size in the male brain following experimental ischemia while having no effect in the female. It implicates neuronal TRPM2 involves in an essential mechanism in experimental stroke [21]. Furthermore, Shimizu et al. have revealed mechanistic insight into the sex-specific role of TRPM2 in neuronal injury after experimental stroke [22]. The proposed lack of neuroprotection effects by clotrimazole in PARP-1 knockout mice, indicating that PARP-1 activity is upstream of TRPM2 activation. Further evidence demonstrated that male cell death after cerebral ischemia was mediated predominantly by excessive ROS production and subsequent overactivation of PARP-1 and TRPM2, eventually resulting in mitochondrial dysfunction, the release of apoptosis-inducing factor, and cell death. In contrast, in females, cell death involves caspase-dependent apoptosis [51–53]. Therefore, it shows that PARP-1-mediated TRPM2 activation is particularly relevant in male ischemic cell death.

Ischemia-induced brain damage is profoundly related to excessive ROS generation [54–56]. Therefore, ROS-induced TRPM2-dependent delayed neuronal cell death may represent a common mechanism in ischemic stroke. As is well known, nitrogen oxides (NOX)‐mediated ROS generation is a vital signaling pathway in ROS‐induced neuronal cell death [57]. H_2_O_2_-induced lysosomal dysfunction also resulted in mitochondrial Zn^2+^ accumulation, fragmentation, and ROS generation that were inhibited by PJ34 or 2-APB (TRPM2 inhibitor), suggesting that these mitochondrial events are TRPM2 dependent and sequela of lysosomal dysfunction. It has well been proved that inhibition of protein kinase C(PKC)/NOX prevented TRPM2-induced delayed neuronal cell death cascade [58]. Futhermore, the recent study also revealed that TRPM2 activation resulted in the production of mitochondrial membrane depolarization-induced free oxygen radical, Ca^2+^ influx, apoptotic factors release (including caspases 3 and 9), and eventual cell death in cerebral ischemia-induced brain hippocampal neuronal injury [59]. These results support an important role for TRPM2 in coupling PKC/NOX-mediated ROS generation, which causes subsequent positive-feedback loops for ROS-induced delayed cell death. Currently, many antioxidants, such as edaravone and *N*-acetylcysteine (NAC), protect nerve cells against cerebral ischemia injury [60]. One study has also shown that treating with edaravone suppresses oxidative stress and axonal injury [61]. The clinical use of edaravone is well established and has led to satisfying outcomes in cerebral infarction [62]. What is more, NAC effectively blocks TRPM2-mediated Ca^2+^ influx, decreases intracellular Ca^2+^ overload, and increases neuronal survival [63]. Therefore, an antioxidant compound that scavenges ROS could play an important role in the treatment of ischemic stroke.

In addition, some studies have shown that downstream signaling molecules such as *N*-methyl-D-aspartate receptors (NMDARs) are associated with TRPM2 activation. NMDARs are mainly composed of a variety of NMDA Receptor 2 (GluN2) A, B, C, and D subunits. GluN2A/GluN2B ratio regulation is a dual rule for TRPM2 in switching from either survival mechanism or cell death [18]. Researchers found that H_2_O_2_ increased synaptic excitability in CA1 neurons from TRPM2^−/-^ but not Wide type (WT) neurons. The increase in excitability resulted from a reduction in GluN2B and an increase in GluN2A expression levels in TRPM2^−/-^ mouse neurons. The changes of GluN2A/GluN2B ratios affect downstream Protein Kinase B (Akt) and extracellular signal-regulated kinase (ERK) pathways leading to a promotion of pro-death and inhibition of pro-survival mechanisms [18,64]. They also demonstrated that, in TRPM2^−/-^ hippocampus, there was a reduction in postsynaptic density-95 kDa (PSD-95) and an increase in phosphorylation of glycogen synthase kinase-3 beta (GSK3β). Furthermore, the expression of TRPM2 is required to promote the expression of PSD-95 and inhibit the GluN2A subunit. PSD-95 is responsible for activating GluN2B. When PSD95 activates GluN2B-containing NMDAR, there is a subsequent influx and accumulation of calcium, which inhibits phosphorylation of protein kinases 1/2 (ERK1/2) and promotes cell death. While inhibiting GluN2A expression reduces synaptic Ca^2+^ influx and prevents downstream activation of MEK and PI3 kinases required for phosphorylation of ERK1/2 and Akt. Phosphorylation of Akt inhibits proapoptotic factor GSK3β. Taken together, TRPM2 modulates NMDAR-dependent survival and death signal pathways [18].

As is well known, Zn^2+^-induced neuronal death leads to Zn^2+^ overload, including lysosomal and mitochondrial dysfunction [65–70]. Ischemic stroke-induced TRPM2 activation leads to extracellular zinc ions significantly increased. Additionally, TRPM2 channel’s genetic deletion prevents increase in Zn^2+^, lysosomal dysfunction, and neuronal cell death induced by H_2_O_2_ [23]. Interestingly, inhibition of such Zn^2+^ signaling significantly attenuates ROS-induced neuronal death [71]. These data show that a significant role of TRPM2 in the intracellular Zn^2+^ homeostasis, lysosomal, and mitochondrial functions in ROS-induced neuronal death.

The nucleotide-binding domain (NBD) and leucine-rich repeat (LRR)-containing protein (NLR) family pyrin domain (PYD)-containing protein 3 (NLRP3) inflammasome can sense a variety of pathogens, further leads to the secretion of pro-inflammatory cytokines and inflammatory cell death. Pan et al. have reported that TRPM2 knockdown reduced OGD-induced neuronal injury, potentially by inhibiting apoptosis and reducing oxidative stress levels, mitochondrial membrane potentials, intracellular calcium concentrations, and NLRP3 inflammasome activation [72]. Upon NLRP3 inflammasome activation, this complex induces cleavage of the procaspase-1. Caspase-1 can cleave the interleukin-18 and interleukin-1β precursors into their active forms, which produces pro-inflammatory effects [73]. Meanwhile, C-X-C motif chemokine ligand 2 (CXCL2) is an inflammatory chemokine, and its corresponding receptor CXC chemokine receptor 2 (CXCR2) is predominantly expressed on the surface of inflammatory cells [74]. The binding of CXCL2 to CXCR2 enhances its expression, induces the migration of neutrophils, dendritic cells, and other inflammatory cells and exacerbates ischemic brain injury [75]. Pan et al. have also revealed that CXCL2, NLRP3, caspase-1 expressions were elevated exposed to OGD, which were attenuated by TRPM2 deletion [72]. Therefore, it is possible that TRPM2 contributes to NLRP3 activation and cell death, although the roles need to be demonstrated by more studies.

**Figure 3.**
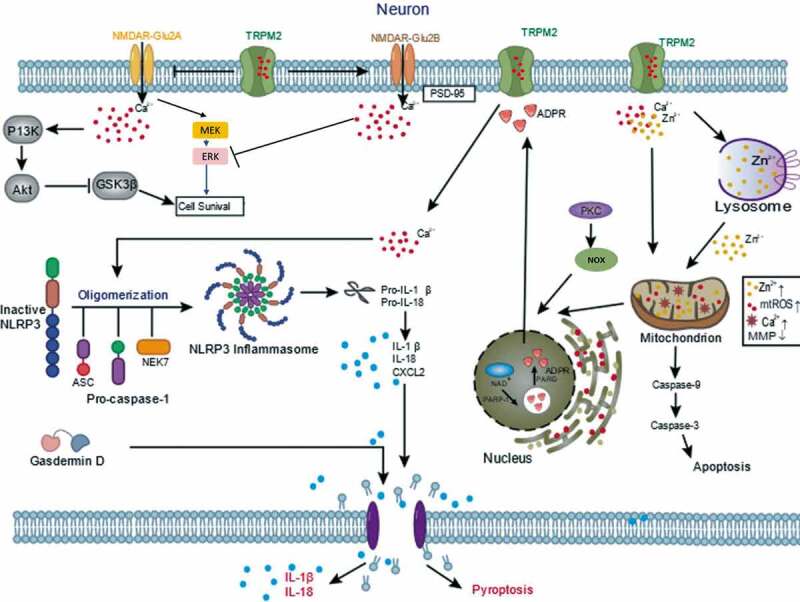
Mechanisms of neuronal TRPM2 in ischemic stroke. TRPM2 modulates NMDAR-dependent survival and death signal pathways. TRPM2 participate in PKC/NOX‐mediated ROS generation, Zn^2+^ accumulation and subsequent a vicious positive feedback signaling mechanism for delayed cell death. TRPM2 involves in NLRP3 inflammasome activation and secretion of CXCL2 and caspase-1

Of note, no difference in infarct volume was observed between TRPM2-KnockOut (TRPM2-KO) and WT mice after permanent ischemia without reperfusion. Such an exclusive role of TRPM2 during reperfusion indicates that TRPM2 deficiency can only protect against brain damage induced by transient (followed by reperfusion) but not permanent (no reperfusion) [20]. The study also investigated that ischemia-induced memory deficits were mediated by the aberrant activity of TRPM2-CaN-GSK3β signaling cascade that actively inhibits synaptic plasticity [76]. Currently, mounting evidence suggested that TRPM2 worked together with diverse mechanisms, contributing to ROS‐induced neuronal cell death. These studies are beneficial to examine neuronal cell death-related with ischemic stroke brain damage (Figure 3).

### Involvement of non-neuronal TRPM2 in immune responses

Emerging studies suggest that ischemic stroke causes inflammation, which leads to increased infarct volume and secondary brain damage [77]. In the transient middle cerebral artery occlusion (t-MCAO) model, oxidative stress promotes the inflammatory processes, which includes infiltration of peripheral immune cells into the CNS and increased activation of glial cells [78]. Of note, TRPM2 control immune cell function and responses, including the production of cytokines and chemotaxis of immune cells, and inflammasome activation [79–82]. For example, increased TRPM2 expression in microglia has been demonstrated weeks after focal cerebral ischemia [28]. The study has revealed that TRPM2-mediated Ca^2+^currents could be detected in cultured microglia [83]. Oxidative stress induces microglial cell activation and neuroinflammation in ischemic stroke. Lee et al. have proposed that TRPM2 produced diverse pro-inflammatory mediators such as TNF-α and interleukin-6 (IL-6) in cultured human microglial cells under buthionine–sulfoximine (BSO)-induced oxidative stress [30]. Also, excessive Ca^2+^ induced by TRPM2 was sufficient to activate mitogen-activated protein kinases (MAPK), p38, extracellular signal-regulated kinase ERK, and Jun-N-terminal kinase (JNK), and downstream nuclear factor NF-kappa B (NF-κB). In comparison, BSO-induced increase in Ca^2+^ and activation of MAPK and NF-κB signaling pathways were profoundly suppressed by using TRPM2 inhibitors.

Apart from the above, TRPM2 played a prominent role in the production of nitric oxide (NO) in microglial cells. Microglial cells undergo multiple morphological and functional changes from the resting cell toward a fully activated, phagocyting tissue macrophage responds to ischemic stroke. Bacterial lipopolysaccharide (LPS) and interferonγ (IFN γ) is a frequently used tool to induce microglia activation. Miyake et al. also recently investigated (LPS/IFNγ) mediated activation of microglia resulted in the induction of TRPM2-mediated Ca^2+^signaling, in turn, led to Pyk2 activation and increases in downstream MAPK and JNK signaling. These intracellular changes lead to inducible nitric oxide synthase (iNOS) and CXCL-2 mRNA upregulation in microglia [84]. Zhu et al. have revealed that TRPM2 played an essential role as an oxidative stress sensor in astrocytes. They found that TRPM2-deficient astrocytes upon LPS stimulation also decreased inflammation mediators (interleukin (IL)-1β, IL-6, and TNF-α level) [85]. Currently, increasing evidence suggested that TRPM2-mediated neuroinflammation.

**Figure 4.**
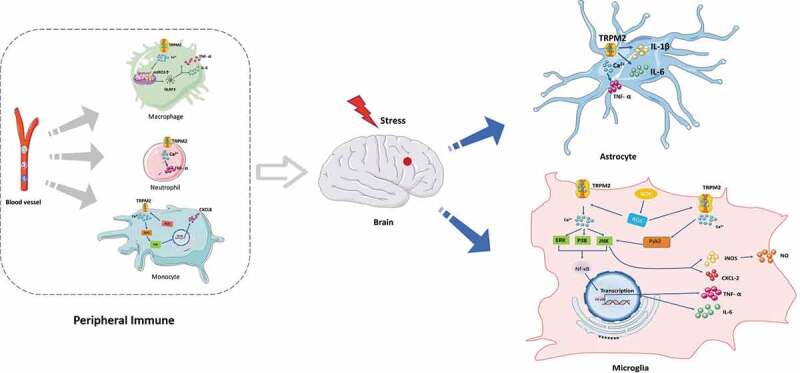
Involvement of non-neuronal TRPM2 in immune responses. TRPM2 signaling control microglia cells and astrocytes function and responses through production of cytokines and chemotaxis. TRPM2 contributes to brain injury through activating peripheral immune cells including macrophages, neutrophils, and monocytes

In addition, it has been shown that TRPM2 contributes to ischemic brain injury after stroke, which mainly depends on its role in activating peripheral immune cells. In vivo experiments with bone marrow chimeric mice show that TRPM2 directly contributes to the migration of neutrophils and, to a lesser extent, of macrophages into the ischemic hemispheres and that TRPM2 in these cell types secondarily aggravates brain damage. TRPM2 deficiency reduces TNF-α secretion in the macrophages, neutrophils, and dendritic cells after ischemic brain injury [86]. Also, loss of TRPM2 attenuates zymosan-evoked macrophage functions, including cytokine release and fever-enhanced phagocytic activity [87]. On the other hand, in human U937 monocytes, Yamamoto et al. revealed that H_2_O_2_ promoted Ca^2+^ influx through TRPM2, which activated Ca^2+^-dependent proline-rich tyrosine kinase 2 (Pyk2) and ERK signaling. It caused the nuclear translocation of Nf-κB, which produced the CXCL8. Conversely, TRPM2-silencing induced the opposite effects in monocytes [82]. ROS production and intracellular calcium can also lead to the activation of NLRP3 [81]. Recent studies have shown that TRPM2-mediated ROS-dependent inflammasome activation in immune cells. More importantly, knockout or inhibition of TRPM2 reversed ROS-dependent NLRP3 inflammasome activation in macrophages [81]. Taken together, TRPM2 can exacerbate systemic immune response in ischemic stroke (Figure 4).

### The role of TRPM2 in blood-brain barrier damage

**Figure 5.**
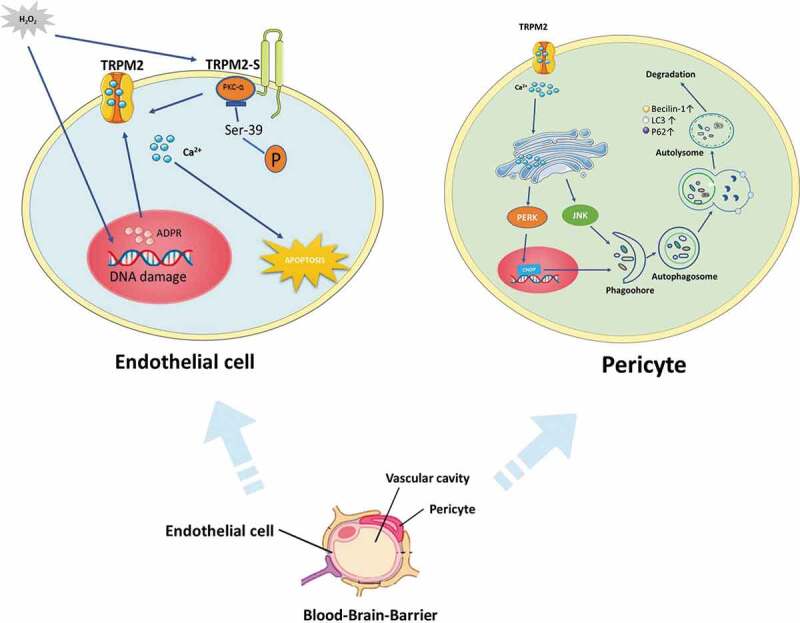
The role of TRPM2 in blood–brain barrier damage. TRPM2 is involved in endothelial cell damage and microvascular pericyte injury

The tight junctions of endothelial cells maintain the low permeability and high transendothelial electrical resistance of the BBB [31]. The neurovascular pericytes are critical components of the BBB [88–90]. A recent study demonstrated that pericytes played a prominent role in the maintenance of the BBB in ischemic stroke [91]. Using the ZnO-NP-induced oxidative stress model in vitro, in combination with genetic and pharmacological approaches, they investigated the role of TRPM2 in the crosstalk that couple autophagy and microvascular pericyte injury [33]. Accordingly, LC3-II accumulation is reduced and pericytes are better preserved in intact brain microvessels of TRPM2 KO mice following ZnO-NP-induced vascular injury. Moreover, TRPM2 lies upstream of the endoplasmic reticulum stress (ER stress)-autophagy axis, and autophagy is functioning primarily as a cytotoxic response to excess ER stress [33]. It is also known that BBB is formed by the endothelial cells of cerebral microvessels, providing a highly selective vascular permeability. Endothelial cell apoptosis can lead to disruption of the endothelial barrier and to inflammation [92]. Activated endothelial cells by generated oxidants are known to induce apoptosis, apathogenic feature of vascular injury and inflammation from ischemic stroke. The study has reported that activation of TRPM2 induced apoptosis of endothelial cells. The signaling mechanism involves ROS-induced PKCα activation resulting in phosphorylation of TRPM2-S at Ser 39 that causes TRPM2-mediated gating of Ca^2+^ influx, which in turn activates caspase-3 and the down-stream apoptosis program [93]. The distinctive mechanism of TRPM2 activation is regarded to be essential in the induction of oxidant-mediated apoptosis of endothelial cells. Therefore, strategies aimed at inhibition of TRPM2 may attenuate endothelial apoptosis and subsequent vascular inflammation injury (Figure 5).

## TRPM2 inhibitors: Potential molecular targeted candidates for ischemic stroke

Studies have demonstrated that TRPM2 contributed to ischemic brain injury. Thus, regulating TRPM2 activation at the molecular level may provide insights into developing new therapeutics for ischemic stroke. It is worth noting that some TRPM2 inhibitors (e.g., ACA, econazole, clotrimazole, and flufenamic acid) show different TRPM2 inhibitory activities. However, with the broad spectrum of their targets, these inhibitors might have substantial side effects. Additionally, there are also several designed and synthesized analogs of endogenous ligands (ADPR), such as 8-Br- ADPR [94], adenosine monophosphate (AMP) [95]. These compounds selectively inhibit TRPM2 currents by binding with NUDT9-H domain or MHR1/2 sites [96]. But most of these inhibitors have poor membrane permeability [38,97]. The peptide inhibitor tat-M2NX also profoundly inhibits ADPR-induced TRPM2 currents, which can interact with the NUDT9-H domain. Moreover, tat-M2NX can efficiently cross the BBB. However, tat-M2NX is to be poorly immunogenic and unsuitable for oral administration [98]. In recent years, High-throughput screening (HTS) is growing rapidly. With this method, scalaradial and JNJ-28,583,113 were found to be effective TRPM2 inhibitors [99,100]. Furthermore, ligand-based drug design (LBDD) depends on pharmacophore modeling validation with low energy to the synthetic structurally diverse compound because the ligands are prone to have similar properties. Thus, pharmacophore docking methods provide guidance for subsequent structural optimization. In the study, the optimized conformation of AMP was used as the basis structure to discover the 2,3-dihydroquinazolin-4 (1 H)-one derivative as a potent TRPM2 inhibitor. The authors used calcium imaging and electrophysiology approaches to evaluate the selected compounds’ inhibitory activities. They also reported synthetic methods and biological characteristics of these compounds, facilitating the discovery of new and promising specific TRPM2 inhibitors [101]. Therefore, the combination of in vitro experiments and ligand-based virtual screening represents a reasonable approach to validate novel candidate compounds as inhibitors of TRPM2 in advance of compound optimization. Such synthesis inhibitors mentioned above play an essential role in experimental trials and may offer new therapeutic strategies for ischemic stroke. These novel synthesis TRPM2 inhibitors are summarized in Table 1.

**Table 1.** Chemical synthesis compounds with TRPM2 inhibitory effects

Compound name	Structure	Animal/Cell	Mechanism of action	References
Flufenamic acid (FFA)		TRPM2-HEK293 cells/Rat insulinoma CRI-G1 cells	Partially reversible and voltage-independence on the inhibition of TRPM2 intracellularly Nonspecifically inhibits TRPM2 and activates TRPC6 and TRPA	[107–109]
2-(3-Methylphenyl) aminobenzoic acid (3-MFA)		TRPM2-HEK293 cells	Partially reversible and voltage-independence on the inhibition of TRPM2 intracellularly Nonspecific block of TRPM2 and slight inhibits the TRPC4 and TRPC5	[108,110]
*N*-(*p*-Amylcinnamoyl) anthranilic acid (ACA)		TRPM2-HEK293 cells/TRPM2-Human U937 cells	Partially reversible and voltage-independence on the inhibition of TRPM2 extracellularly Nonspecifically blocks TRPM2 and inhibits the other TRP channels in the order: TRPM8 > TRPC6 > TRPV1 Decreases the male hippocampal cell death and infarct size in ischemic stroke Suppresses neutrophil infiltration after ischemic stroke Attenuates okadaic acid-induced neuroinflammation and neurodegeneration in rats	[19,21,86,87,111–113]
Clotrimazole (Cltz)		TRPM2-HEK293 cells/Rat insulinoma CRI-G1 cells	Completely irreversible inhibition of TRPM2 and accelerates the inhibition effects as extracellular pH falls Nonselectively blocks TRPM2 and displays concentration dependence Decreases hippocampal cell death and infarct volume following cerebral ischemia	[19,22,53,114]
Econazole		TRPM2-HEK293 cells	Irreversible inhibition of TRPM2-mediated currents in a concentration-dependent manner Nonselective inhibition of TRPM2 by altering cytosolic Ca^2+^ concentration	[114]
2-Aminoethoxydiphenyl borate (2-APB)		TRPM2-HEK293 cells	Voltage independent but reversibly inhibition of TRPM2 extracellularly Nonselectively blocks TRPM2 by altering cytosolic Ca^2+^ accumulation Protects kidney function and decreases neutrophil infiltration after kidney I/R injury Decreases male hippocampal cell death following OGD Attenuates diabetes-induced cognitive dysfunction	[19,53,108,115,116]
Adenosine monophosphate (AMP)		Human neutrophils and T cells	Potent dose-dependent inhibition of ADPR-induced TRPM2 currents Specifically inhibits TRPM2 currents at the NUDIX domain	[95,117]
8-Bromoadenosine 5′-diphosphoribose (8‐Br‐cADPR)		Male Wistar albino rats	Completely suppressed cADPR and H_2_O_2_-induced TRPM2 currents but not ADPR-induced TRPM2 currents Specifically inhibits TRPM2 currents at the MHR1/2 domain Reduces renal damage after renal ischemia–reperfusion injury Decreases expressions of TRPM2, CD38, caspase‐3, TNF-α, IL‐1β	[94,95,117–119]
8-Phenyl-2′-deoxy- ADPR (8-Ph-ADPR)		TRPM2-HEK293 cells	Highly active specific NUDT9H- TRPM2 antagonist without affecting TRPM7, TRPM8 Inhibition of Ca^2+^ signaling and chemotaxis in human neutrophils	[118,120]
Tat-M2NX	/	C57Bl/6 mice	Specifically inhibits ADPR-induced TRPM2 currents involved direct interactions with the NUDT9-H domain Reduction in infarct volume after ischemic stroke injury Efficiently cross the BBB	[98]
Scalaradial		TRPM2- HEK293 cells	Inhibition of TRPM2 currents in a concentration and time-dependent manner Specifically blocks TRPM2 Independence of phospholipase A2(sPLA2) and ERK and Akt pathways	[99]
JNJ-28,583,113		TRPM2-expressing HEK cells	Reversible inhibition of TRPM2 Causes the phosphorylation of the GSK3α and β subunits in microglia Protected cells from oxidative stress-induced cell death, cellular morphological changes and blunted microglia cytokine release in response to pro-inflammatory stimuli	[100]
7i		TRPM2-HEK293 cells	Dose-dependent inhibition of TRPM2 Interaction with NUDT9-H domain of TRPM2 Selective inhibition of TRPM2 currents without affecting TRPM7, TRPM8, TRPV1, and TRPV3	[120]
8a		TRPM2-HEK293 cells	Dose-dependent inhibition of TRPM2 Interaction with NUDT9-H domain of TRPM2 Selective inhibition of TRPM2 currents without affecting TRPM7, TRPM8, TRPV1, and TRPV3	[120]
2,3-Dihydroquinazolin-4(1 H)-one (D9)		TRPM2-HEK293 cells	Specific inhibitor of TRPM2 without affecting the TRPM8 currents	[101]
*N*-Acetyl-L-cysteine		Rats induced by global cerebral ischemia	Reduction in global cerebral ischemia-induced neuronal death cascades, such as lipid peroxidation, microglia, and astroglia activation, free zinc accumulation, and TRPM2 over-activation	[121]
AG490		TRPM2-HEK293 cells/Primary neurons were cultured from E16 CD-1 mice	Reduction in TRPM2 currents and infarct volume Improvement in overall brain morphology, general health, and short-term neurobehavioral performance	[122]
AG-related compounds (AG555, AG556)	/	Human monocytic U937 cells	Blocks H_2_O_2_-induced TRPM2 activation by scavenging of the hydroxyl radical Inhibits H_2_O_2_-induced CXCL8 secretion following ERK activation, which is mediated by TRPM2-dependent and independent mechanisms in U937 cells	[123]

Compared to synthetic TRPM2 inhibitors, natural inhibitors are preferred for safety reasons. Natural herbal plants have been a source of medicine for many centuries. Due to their advantages, including abundance in nature, multi-target efficacy, limited side effects, and low toxicity, traditional Chinese medicines have attracted increasing attention, specifically those medicines containing active neuroprotective components. It is noteworthy that some active ingredients of traditional Chinese medicines may inhibit TRPM2 through antioxidative effects. For instance, curcumin reduces TRPM2 currents, mitochondria, and DNA damage via inhibiting the production of ADPR as an antioxidant and free-radical scavenger [102]. Besides, many examples of marketed drugs being applied to new diseases suggest that these molecules have numerous varied effects. For example, among the seven antidepressants tested, duloxetine exerts the strongest inhibitory effects on TRPM2 activation. Further, the administration of duloxetine reduces ischemic brain injury. As a result, duloxetine may be a useful drug in ischemic stroke because it has already been used clinically in therapeutics for several disorders, including depression [103]. Therefore, the use of marketed drugs, including TRPM2 inhibitors, can simplify drug development and reduce economic and social costs. TRPM2 inhibitors of natural origin and marketed drugs are summarized in Table 2.

**Table 2.** Literature review of various plants/herbs and marketed drugs showing TRPM2 inhibition effects

Compound name	Structure	Disease	Pharmacological activity	References
Duloxetine		Cerebral I/R injury Depression	Reduction in TRPM2-mediated inward currents during the channel-opening state Reduction in Ca^2+^ concentration, caspase-3, caspase-9, mitochondrial depolarization, and intracellular ROS production Attenuates brain injury via TRPM2 inhibition	[103,124]
Dexmedetomidine		Cerebral ischemia	Reduction in TRPM2 densities and cytosolic calcium ion accumulation Decrease in caspase-3, caspase-9, ROS production, and depolarization of mitochondrial membrane potential in the hippocampus and dorsal root ganglion (DRG) neurons	[59]
Melatonin and selenium		Neuropathic pain	Decrease in Ca^2+^ concentration, caspase-3, caspase-9, the current density in TRPM2 and amelioration of apoptosis and mitochondrial depolarization Reduction of glutathione, lipid peroxidation, and intracellular ROS production Prevents IFNγ-mediated microglia TRPM2 activation and cytokine generation	[125–127]
17β-Estradiol		Neurodegenerative disease in postmenopausal women	Decreases Ca^2**+**^ influx, TRPM2 capacitances, caspase-3, caspase-9, mitochondrial membrane depolarization, and cell viability	[128]
Tamoxifen		Neurodegenerative disease in postmenopausal women	Dcrease in Ca^2+^concentration, TRPM2 capacitance, caspase-3, caspase-9, and mitochondrial depolarization	[128]
Raloxifene		Neurodegenerative disease in postmenopausal women	Decrease in Ca^2**+**^ concentration, TRPM2 current density, caspase-3, caspase-9, and mitochondrial membrane depolarization	[128]
Curcumin		Liver I/R injury Drug-induced hepatitis Nonalcoholic steatohepatitis	Inhibition of TRPM2 currents and increased Ca^2+^ in rat hepatocytes	[102]
		Cisplatin-induced optic nerve damage	Decreases TRPM2 current density, Ca^2+^ concentration, mitochondrial membrane depolarization, and ROS production	[129]
		Chronic kidney disease	Inhibition of albumin-induced Ca^2+^ oscillations, cytokine production, ROS production, mitochondrial membrane potential, and NF-κB signaling related to cell death via TRPM2 inhibition	[130]
Salidroside		Liver disease	Inhibition of the increases in TRPM2 protein and mRNA expressions, Ca^2+^ increase, and p-CaMKII protein expression Increase in the relative expression of LC3B-II and reduction of p62, and LC3B-II/LC3B-I ratio Alleviates PA-induced lipid accumulation, IL-1β and IL-6 mRNA expression in hepatic L02 cells	[131]
Resveratrol		Acute hypoxia	Reduction of intracellular Ca^2+^ and improves mitochondrial function Suppression of the generation of cytokines (IL-1β and TNF-α), Zn^2+^ concentration, PARP-1 activity, TRPM2 expression, caspase-3, and caspase-9 levels, and mitochondrial ROS production in SH-SY5Y cells	[132]
Hypericum perforatum (HP)		Spinal cord injury	Reduction of the SCI-induced TRPM2 currents and cytosolic-free Ca^2+^ concentration Reduces the increase in apoptosis, caspase 3, caspase 9, cytosolic ROS production, and mitochondrial membrane depolarization values in the DRG of the spinal cord injury group	[133]

## Future perspectives for TRPM2 inhibitors

Rational drug design that targets TRPM2 is a promising approach for treating ischemic stroke. Structure-based virtual screening approaches may prove useful in the development of therapeutic agents. Given the high potency and binding affinity of molecular docking, it is essential to consider ligand–receptor interactions, including target structure, ligand-binding properties, and pharmacological activity [104]. Currently, several cryo-EM structures of human TRPM2 in complex with ADPR or 8-Br-cADPR have been resolved, which will provide more precise ligand-binding site information for structure-based virtual screening methods, thereby enabling researchers to design inhibitors targeting TRPM2. There are mainly three binding sites of TRPM2 for inhibitor binding: the extracellular region, and intracellular MHR1/2 and NUDT9-H domains.

However, ligand docking and virtual screening have limitations. The results of structure-based virtual screening can only be considered preliminary and are sometimes not satisfactory [105]. The receptor may be experimentally determined to be unsuitable for docking studies, or differences in the receptor binding sites may be found. Thus, further in vivo and in vitro experiments are required to confirm the virtual screening results. Besides, it is important to assess the safety and efficacy of candidate compounds before clinical application. The future direction of novel TRPM2 inhibitors depends on their targeting specificity. The solution of treatment for cerebral ischemia may require multidisciplinary research involving fields such as medicinal/pharmaceutical chemistry, bioinformatics, biochemistry, proteomics, and metabolomics, which have all contributed to the rational drug design of TRPM2 ion-channel inhibitors [106]. Accordingly, the development of specific TRPM2 inhibitors can provide insights for therapeutic intervention in ischemic stroke.

## Summary

Despite advances in the understanding of the pathogenesis of cerebral ischemia, there is still a lack of effective and potent therapeutic options. Mounting evidence show that TRPM2 interacts with multiple regulatory pathways in neurons, glia, and cells of immune system and BBB, which leads to severe brain injury after ischemia. These studies indicate that TRPM2 is a promising target for improvement of ischemic stroke. However, the precise molecular mechanisms involved in TRPM2-mediated ischemic stroke remain completely unknown. Therefore, a better understanding of the damage mechanisms at the molecular levels is helpful for the development of novel therapeutic strategies of targeting TRPM2 to treat ischemic brain injury. In addition, available pharmacologic TRPM2 inhibitors, unfortunately, lack specificity. The inhibitory mechanisms and binding sites of most reported TRPM2 inhibitors are still unclear. Hence, there is an urgent need to develop effective and specific neuroprotective agents targeting TRPM2 for attenuating ischemic stroke.
